# Acetylcholinesterase Activity Influenced by Lipid Membrane Area and Surface Acoustic Waves

**DOI:** 10.3390/mi13020287

**Published:** 2022-02-11

**Authors:** Lukas G. Schnitzler, Kathrin Baumgartner, Anna Kolb, Benedikt Braun, Christoph Westerhausen

**Affiliations:** 1 Experimental Physics I, Institute of Physics, University of Augsburg, 86159 Augsburg, Germany; lukas.schnitzler@physik.uni-augsburg.de (L.G.S.); kathrin.baumgartner@med.uni-augsburg.de (K.B.); anna.kolb@kabelmail.de (A.K.); benedikt.braun@uni-a.de (B.B.); 2 Center for NanoScience (CeNS), Ludwig-Maximilians-Universität Munich, 80799 Munich, Germany; 3 Physiology, Institute of Theoretical Medicine, University of Augsburg, 86159 Augsburg, Germany; 4 Augsburg Center for Innovative Technologies (ACIT), 86159 Augsburg, Germany

**Keywords:** acetylcholinesterase, enzyme activity, Ellman assay, lipid membrane, surface acoustic waves

## Abstract

According to the current model of nerve propagation, the function of acetylcholinesterase (AChE) is to terminate synaptic transmission of nerve signals by hydrolyzing the neurotransmitter acetylcholine (ACh) in the synaptic cleft to acetic acid (acetate) and choline. However, extra-synaptic roles, which are known as ‘non-classical’ roles, have not been fully elucidated. Here, we measured AChE activity with the enzyme bound to lipid membranes of varying area per enzyme in vitro using the Ellman assay. We found that the activity was not affected by density fluctuations in a supported lipid bilayer (SLB) induced by standing surface acoustic waves. Nevertheless, we found twice as high activity in the presence of small unilamellar vesicles (SUV) compared to lipid-free samples. We also showed that the increase in activity scaled with the available membrane area per enzyme.

## 1. Introduction

The lateral organization of the cell membrane, including so-called lipid rafts, is essential for the formation of functional units in biology [1,2,3,4]. Although largely unknown, these domains are critical for biological signal transduction, enzyme catalysis, or receptor mobility. A simple approach to mimic rafts could be based on patterning membranes. There exist several techniques to induce patterns in solid-state supported lipid bilayers (SLB). Groves et al. lithographically patterned grids of photoresist, aluminum oxide, or gold on oxidized silicon substrates to partition supported lipid bilayers into micrometer-scale arrays of isolated fluid membrane corrals. Application of an electric field parallel to the surface induces steady-state concentration gradients of charged membrane components in the corrals [5,6,7,8]. Moreover, Hochrein et al. studied the conformational behaviour of DNA molecules adsorbed on cationic lipid membranes deposited on grooved, one-dimensional, periodic, microstructured surfaces [9]. Another patterning technique uses proteins and PDMS stamps to induce patterns in SLB [10]. Sanni et al. combined hierarchical surface wrinkling of elastomers with lipid membrane deposition techniques to dynamically template complex three-dimensional topographies onto supported lipid bilayers [11]. Techniques to actively manipulate and control the lateral dynamics of the membrane, however, are still lacking. This apparent shortcoming was pointed out by Jacobson et al.: “However, the field of lipid rafts is currently at a technical impasse, as the physical tools to study biological membranes as spatially and temporally ordered fluid are still being developed.” [4] Surface acoustic waves (SAW) with amplitudes of the order of 1 nm and variable wavelength and frequency between about 30 μm at 100 MHz and 3 μm at 1000 MHz can be used to generate standing waves and thus a tunable energy landscape on a chip. As previously shown, it is possible to actively control the spatial and temporal organization of a SLB thereby [12,13]. Furthermore, molecules such as dyes or proteins (e.g., receptors or enzymes) incorporated into the SLB can be organized both selectively and reversibly into stripe- and dot-like clusters [12,13,14,15]. Thus, SAW technology could offer the possibility of forming lipid rafts, or more general coexistence of domains of different lipid order and composition as present in the proximity of phase transitions, and thus mimicking biological processes. To test this idea here, we incorporated acetylcholinesterase (AChE) into lipid bilayers and investigated the influence of such density modulations induced by SAW. AChE is one of the fastest enzymes in biological processes. It is found mainly in neuromuscular junctions and in cholinergic synapses. According to the present model of nerve propagation, its function is to terminate synaptic transmission of nerve propagation signals by hydrolyzing the neurotransmitter acetylcholine (ACh) into acetic acid (acetate) and choline within the synaptic cleft. For example, the most prominent function of AChE, catalytic activity, has been shown to be a membrane-mediated function. In addition, the possible biophysical and biological consequences of the rapid hydrolysis of acetylcholine generating high local proton concentrations has been discussed recently [16]. Back in the 1970s, many studies reported deviations from a pure Arrhenius behavior: kinks and non-linearities in the enzyme activity as functions of temperature have been shown in reviews, for instance by Sandermann [17]. Within the last ten years, Schneider and coworkers reported that the activity of AChE and other enzymes shows a pronounced maximum close to the main phase transition of lipid interfaces [18,19]. This does not only hold for quasi-static systems, but also for dynamical ones, i.e., applied pH pulses to 1,2-dimyristoyl-sn-glycero-3-phospho-L-serine (DMPS) monolayers [20]. Recently, we have shown that this is also the case for originally water-soluble enzymes when bound to a lipid bilayer [21]. One theory to explain this effect is based on higher fluctuations that occur in the system within a phase transition [22], based on the original theory of Kaufmann [23]. These results make it seem plausible that artificial changes in the membrane environment, such as density modulations, affect the activity of AChE. Thus, AChE is an ideal choice to study the influence of artificial changes of the lipid membrane on enzyme activity.

## 2. Materials and Methods

### 2.1. Vesicle Fusion

The vesicle fusion method is used to prepare a SLB [24,25,26,27,28,29,30]. In the first step, 1 mg of 1,2-dioleoyl-sn-glycero-3-phosphocholine (DOPC) (Avanti Polar Lipids Inc., Alabastar, AL, USA) is dried with 0.25 mol% DiOC14(3) hydroxyethanesulfonate (DiOC14) (Biotium Inc, Fremont, CA, USA) overnight under vacuum confinement in a glass container. The lipids are then redissolved in 1 mL ultrapure water and mulitlamellar vesicles (MLV) are prepared. For this purpose, the glass container with the lipids is heated to 50 °C for 2 h in a water bath. Every 30 min, the glass container is removed from the water bath and shaken. Alternatively, the glass container can also be placed in an ultrasonic bath for 2 h. The MLV are then further processed to small unilamellar vesicles (SUV) in a subsequent step. The sample is treated with a trunk sonicator (Sonoplus UW 2070, BANDELIN electronic, Berlin, Germany) for 10 min (65% intensity, 50% cycle). Afterwards, the sample is centrifuged (10 min, 14,500 rpm) and the supernatant is collected. Alternatively, the SUV can be prepared using a vial tweeter (UIS250v, Hielscher Ultrasonics GmbH, Teltow, Deutschland) (10 min, 65% intensity, 50% cycle). As a control, the size of the resulting SUV is determined using dynamic light scattering (DLS) (90Plus Particle Size Analyzer, Brookhaven Instruments Corporation, New York, NY, USA). The hydrodynamic diameter should not be larger than 100 nm. Prior to use, the substrates are carefully treated with a cleaning routine. First, the substrates are rinsed with ethanol. Then, the substrates are stored in a 1% solution of Mucasol (Merz Consumer Care GmbH, Frankfurt, Germany) for 10 min. Afterwards, the substrates are placed in an ultrasonic bath for 5 min and rinsed intensively with water. Finally, for hydrophilization, the substrates are exposed to an oxygen plasma (300 Autoload-PC Plasma Processor, Technics Plasma GmbH, Wettenberg, Germany). The final SUV solution is then placed on the hydrophilized substrate in a PDMS (Sylgard 184 Silicone Elastomer, Dow corning, Midland, MI, USA) reservoir. After 30 min, the excess SUV are removed by rinsing several times with ultrapure water.

### 2.2. Ellman Assay

In aqueous solution, the enzyme AChE accelerates the cleavage of acetylthiocholine (ASCh) to thiocholine (SCh) and acetate with the release of protons AChE acts as a catalyst here. The enzyme activity of AChE can be measured using the so-called Ellman assay [31,32]. Here, the dye 5,5′-dithiobis-2-nitrobenzoic acid (DTNB), also called Ellman reagent, is added to the reaction. DTNB reacts with the ASCh formed in the reaction to form a dianion. The dianion shows strong absorption of light at about 412 nm, so it appears yellow. By measuring the absorbance, the increase in dye concentration can be detected. From the slope of the trace, the enzyme activity can be determined by subtracting the thermal decay of ASCh. If not mentioned otherwise, the final concentrations used in the experiment were 0.1 mM DTNB (5,5’-dithiobis-2- nitrobenzoic acid, Sigma-Aldrich, St. Louis, MO, USA), 2 mM ASCh (acetylthiocholine iodide, Sigma-Aldrich, St. Louis, MO, USA) and varying enzyme concentrations between 0.05 nM and 0.8 nM. All samples are prepared in 20 mM HEPES buffer solution (HEPES, sodium salt, EMD Biosciences Inc, La Jolla, CA, USA) at pH 7. The enzyme was always added immediately prior to measuring the samples. In most of the experiments the absorbance is measured with a Plate reader (Infinite 200 PRO, Tecan, Männedorf, Switzerland) at 415 nm in transparent well plates (96-well Clear Polystyrene Microplates, Corning, Corning, NY, USA). The measurements under influence of a SAW standing wave field were performed with an inverted light microscope (Zeiss Axiovert 200M, Carl Zeiss AG, Oberkochen, Germany) where a bandpass filter (FB410-10, Thorlabs Inc, Newton, NJ, USA) was placed in the light path. The intensity is recorded with a CCD-camera (ORCA-05G, Hamamatsu Photonics, Hamamatsu, Japan). The measurements under influence of an electric field were performed with a self-made setup consisting of a LED (LED405E, Thorlabs Inc, Newton, NJ, USA) as a light source, a bandpass filter (FB410-10, Thorlabs Inc., Newton, NJ, USA) and a photo detector (DET100A2, Thorlabs Inc, Newton, NJ, USA). The electric signal is generated with a signal generator (Agilent 33250A, Agilent Technologies, Santa Clara, CA, USA). The absorbance A can be calculated as follows:

(1) $$A=\log\left(\frac{I_{0}-I_{b}}{I-I_{b}}\right)$$

where $I_{0}$ is intensity of the light source, $I_{b}$ is the dark current of the detector or camera and I is the measured intensity after passing the sample.

### 2.3. Simulation of Lipid Diffusion

To simulate the decay of the pattern in a SLB, a random walk algorithm with fixed grid points was used. At each time step, each particle can randomly move to a neighboring grid point and change position with that neighboring particle. However, this is only allowed if the neighboring particle or the particle itself has not yet changed its position in that time step. A periodic boundary condition is applied to the edges of the lattice. The time step dt is defined by $\mathrm{dt}=\frac{{\mathrm{dx}}^{2}}{4D}\;$, where D is the diffusion constant and  dx is the step size of the lattice. At t=0 the following intensity profile is given, which represents the striped pattern observed in the experiment:

(2) $$I=\;a+b\;\exp\left[-{\mathrm{csin}}^{2}\left(k\left(x-d\right)\right)\right]$$

### 2.4. SAW Chip

A pair of interdigital transducers (IDT) of Ti-Au-Ti (5 nm, 50 nm, 5 nm height), aligned along the main propagation direction (X-direction) of the LiTaO_3_ 36° Y cut generates surface acoustic waves (SAW) with a wavelength of $\lambda_{\mathrm{SAW}}=25\;\mu m$ at a sound path width W=1 mm. Each IDT consist of 27 finger pairs. The measured resonant frequency of each IDT is $f_{\mathrm{res}}=163.5\;\mathrm{MHz}$ and the distance between the IDT is l=3.5 mm. To protect the multi-finger electrodes, a SiO_2_ coating was deposited on top of the IDT structures by thermal evaporation. The RF signal is generated and divided by a frequency generator (SML01, Rhode & Schwarz GmbH, Munich, Germany) with an amplifier (gain factor G = 30 dB, AMP590033H-T, Becker Nachrichtentechnik GmbH, Asbach, Germany) and a power splitter (ZFSC-2-4+, Minicircuits, Brooklyn, NY, USA). A PTFE ring (diameter: 1 cm, height: 1 cm) is placed on the SAW chip. To prevent leakage, the bottom of the PTFE ring is sealed with silicone paste (KORASILON-Paste, Kurt Obermeier GmbH & Co. KG, Bad Berleburg, Deutschland).

## 3. Results and Discussion

### 3.1. Bilayer Characterization Using Continuous Bleaching

**Figure 1:**
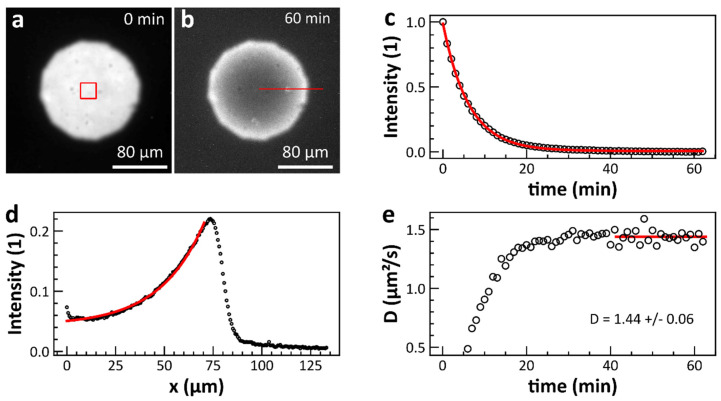
Detection of an SLB by the continuous bleaching method. (**a**,**b**) By continuous excitation of the fluorescent dye incorporated in the lipid membrane, a brightness gradient is obtained over time due to the bleaching and lateral diffusion of the individual lipids. From the bleaching constant of the dye (**c**) and the decay constant of the intensity profile (**d**) along radial directions (averaging the intensity by rotating the line shown in (**b**)), the diffusion constant can be determined. (**e**) Time evolution of the diffusion constant. After complete bleaching in the center, a state of equilibrium is reached, resulting in a constant value for the diffusion constant fitted by the solid line. The circles in c-e represent the single data points. The solid lines are fits to the data according to Equations (3) and (4) in (**c**,**d**).

Before measuring enzyme activity under the influence of surface acoustic waves, we ensured that we could reproducibly generate SLB on SAW chips. SLB were prepared by the so-called vesicle fusion method (for details see materials and methods). In this method, SUV are added in aqueous solution to a hydrophilized substrate. At the substrate surface, the SUV burst and form a closed SLB after a short time. The presence of a SLB can be detected, e.g., using the continuous bleaching method [33,34,35,36]. Therefore, a small amount of fluorescent dye molecules is added to the SLB. Continuous excitation of the fluorescent dye results in an intensity gradient due to bleaching and diffusion in the SLB. To obtain a defined bleaching region, the area of illumination is confined with a spherical aperture (Figure 1a,b).

The bleaching of the dye is an exponential function of time and can be determined in the center of the image (Figure 1c):

(3) $$I\left(t\right)={\;I}_{t0}\exp\left(-B_{0}t\right)+c$$

with $I_{t0}$ the initial intensity and $B_{0}$ the bleaching constant. Furthermore, the intensity profile (Figure 1d) along the line shown in Figure 1b follows:

(4) $$I\left(x\right)={\;I}_{x0}\exp\left(-{\left(B_{0}/D\right)}^{-1/2}x\right)+c$$

with ${\;I}_{x0}$ the intensity at the edge and D the diffusion constant. From the bleaching constant of the dye and the decay constant of the intensity profile, the diffusion constant can be determined. Figure 1e shows the evolution of the diffusion constant for each time step. After a certain time, D(t) approaches a constant value. This value then gives the diffusion constant in the SLB. For a glass substrate, we measured a diffusion constant of about D = 1.6 +/− 0.1 μm^2^/s (n = 3, error represents standard deviation) at 25 °C. This result is consistent with literature values for typical diffusion constants [37]. For the diffusion constant on an SAW chip, we get noticeably lower values of D = 0.18 +/− 0.05 μm^2^/s (n = 3, error represents standard deviation) at 25 °C. This reduction could originate from the difference in surface properties or due to the fact that the SAW chips are reused in the experiment.

### 3.2. Domain Formation by Standing Surface Acoustic Wave

**Figure 2:**
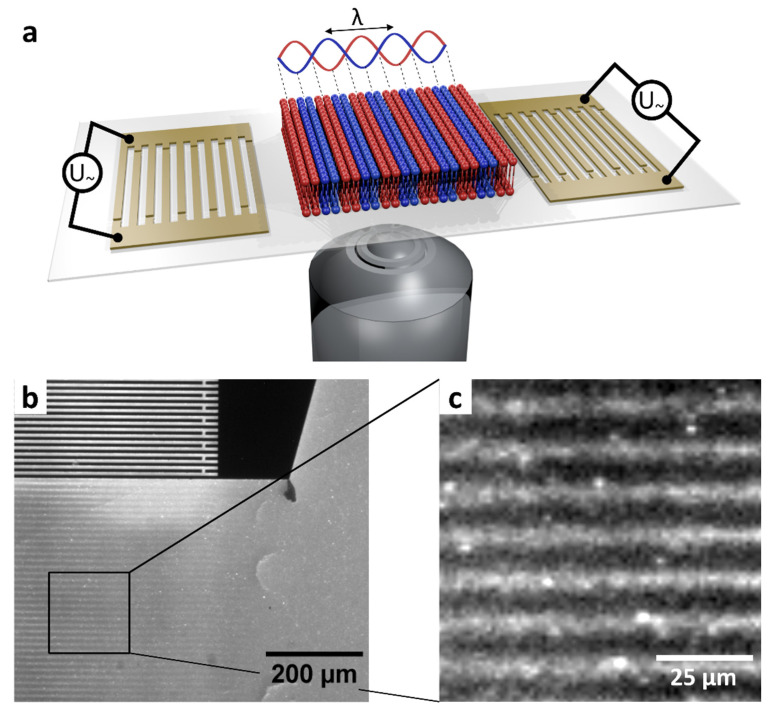
Formation of domains in a SLB of DOPC lipids with 0.25 mol% fluorescent dye DiOC_14_ by applying a SAW standing wave field. (**a**) Sketch of the experimental setup. By applying a RF signal with the resonance frequency f_0_ = 163.5 MHz at two IDT a standing wave is generated, which leads to a density modulation in a SLB. (**b**) The domains are spatially confined to the aperture of the IDT. Outside the excitation region partial extensions of the domains appear. (**c**) Enlarged view of the clearly visible stripe pattern generated by the SAW standing wave field.

After demonstrating the existence of the SLB and the characterization of D, we show here that we can use surface acoustic waves to induce density changes in the lipid membrane. We will use these density modulations later to control the available membrane area per enzyme. As Neumann and Hennig et al. have already shown, a SAW standing wave field modulates the lateral density in an SLB [12,13,14,15]. With a comparable setup (Figure 2a), these results could be reproduced here. Figure 2b shows the formation of domains in a SLB of DOPC lipids with 0.25 mol% DiOC_14_ on a LiTaO_3_ chip. A standing wave is generated by applying a SAW signal to two opposing IDT. This changes the density in the lipid membrane locally in the nodal and anti-nodal regions, As can be seen in Figure 2c, the domains are spatially confined to the aperture of the IDT. The distance between two adjacent nodes matches with the expected value of half a wavelength.

**Figure 3:**
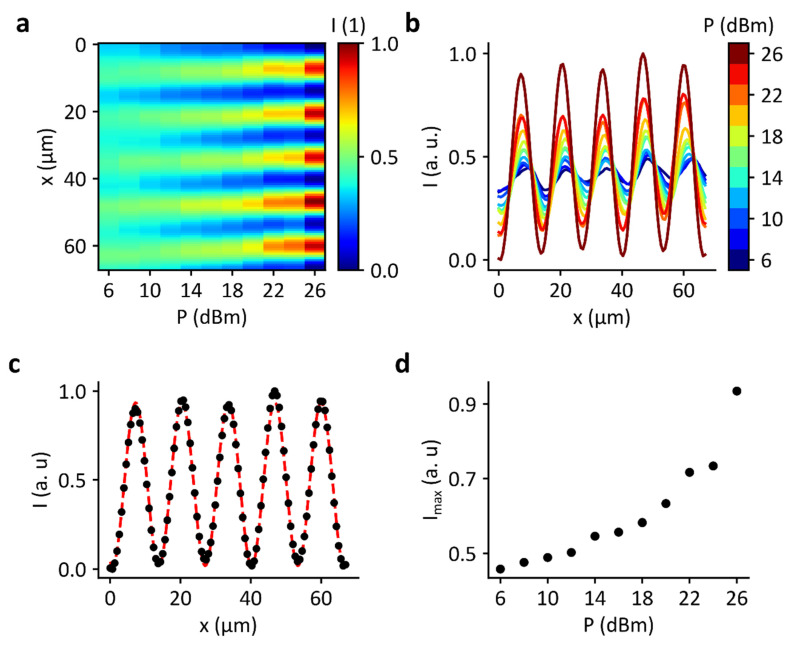
RF power variation of a SAW standing wave field inducing domains in a SLB: (**a**) Heatmap of the normalized brightness intensity in the power range from P_IDT_ = 6 to 26 dBm. (**b**) Intensity profile perpendicular to the pattern of stripes. (**c**) Intensity profile for 26 dBm with a fit function (solid line) according to Equation (5). (**d**) Dependency of the intensity maximum as a function of power. With increasing power, the intensity of the pattern increases.

The influence of the power of the RF signal on the pattern is shown in Figure 3. The power was increased here in 2 dB steps and a fluorescence image was recorded in each case (see Figure 3c). Figure 3a shows the brightness intensity in the image normalized to one for each power. To compensate for the differences in brightness, each image was divided by its mean intensity value. As the power increases, the pattern becomes more pronounced. This means, the difference in brightness between nodes and antinodes increases. This effect is even more evident in Figure 3b. Here, the intensity is shown perpendicular to the stripes. According to Neumann et al. [13], this intensity gradient can be expressed by the following empirical fit function:

(5) $$I=\;a+\mathrm{bexp}\left[-{\mathrm{csin}}^{2}\left(k\left(x-d\right)\right)\right].$$

where a, b, c and d are fit parameters., k corresponds to the wavenumber. Figure 3c shows the experimentally determined intensity curve for 26 dBm with a fit (red line). The fit agrees very well with the measured data. Figure 3d shows the intensity maximum determined from the fits as a function of power. The curve confirms the impression that the brightness or intensity of the stripes increases with increasing power. This result can be explained by the fact that more dye is concentrated in the nodes at higher power.

### 3.3. Influence on Enzyme Activity by Surface Acoustic Waves

**Figure 4:**
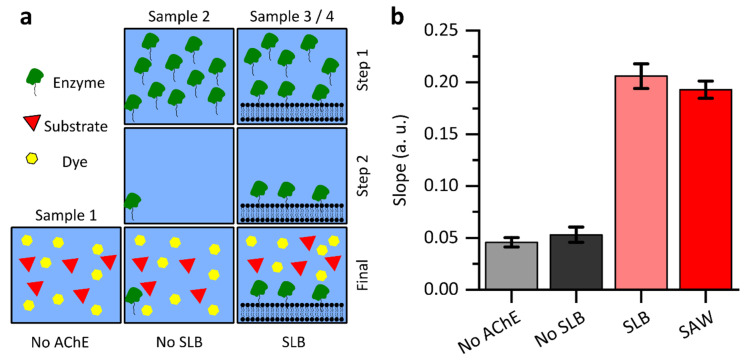
Determination of enzyme binding to SLB and influence of a SAW standing wave field on enzyme activity. (**a**) Preparation steps of the different samples. (**b**) Measurement of absorbance after 1 h by taking out 50 μL. Sample 2 (no SLB) shows nearly the same absorbance as Sample 1 (no AChE). Sample 3 (SLB), on the other hand, shows a much higher absorbance. This suggests that the enzyme is bound to the SLB. Sample 4 (SAW) shows almost the same absorbance as Sample 3 (SLB). Therefore, there is no significant difference due to the SAW treatment. Error bars represent the standard deviation.

In a next step, AChE was embedded into the SLB. With endpoint measurements, we investigated the binding efficiency of the enzyme to the SLB as well as the influence of a SAW standing wave field on the activity of the bound enzyme. Four samples each were prepared for this purpose. Sample 1 contains only dye and substrate and serves as a control for the thermal decay of the substrate. Sample 2 contains no SLB and serves as a control to check whether the enzyme remains in the sample after rinsing even without SLB. Sample 3 contains a SLB but is not treated with SAW. Sample 4 contains a SLB and is treated with SAW (f = 163.5 MHz, P = 12 dBm, t = 1 h). To check whether the enzyme binds to the SLB, the same amount of enzyme (10 μL, c = 4 nM) was added to the samples 2, 3 and 4. After about 30 min, the samples were then rinsed with buffer. Ideally, sample 2 should contain only buffer. Samples 3 and 4 should then contain only enzyme bound to the SLB. Substrate and dye were added last in each case (Figure 4a). After 1 h, 50 μL were taken from each sample and the absorbance was measured with a plate reader. Figure 4b shows the absorbance of the four samples. As expected, the comparison between the sample without the enzyme (Sample 1) and the sample without SLB (Sample 2) show almost the same absorbance. The slightly higher absorbance of Sample 2 can be explained by the fact that a small amount of enzyme remains in the sample after the rinsing step. However, the majority is removed. In comparison, the sample with SLB (Sample 3) shows a significantly higher absorbance. Thus, considerably more product was formed in the same amount of time and is detected by the dye. This indicates that the enzyme is bound to the SLB and is not removed by the rinsing step. Finally, evaluating the absorbance of the SAW-treated sample (Sample 4), we find that it is comparable to that of Sample 3. This indicates that within the uncertainty range here, SAW treatment does not have an effect on enzyme activity in these endpoint measurements. Next, we have a closer look to the enzyme kinetics with and without SAW application.

**Figure 5:**
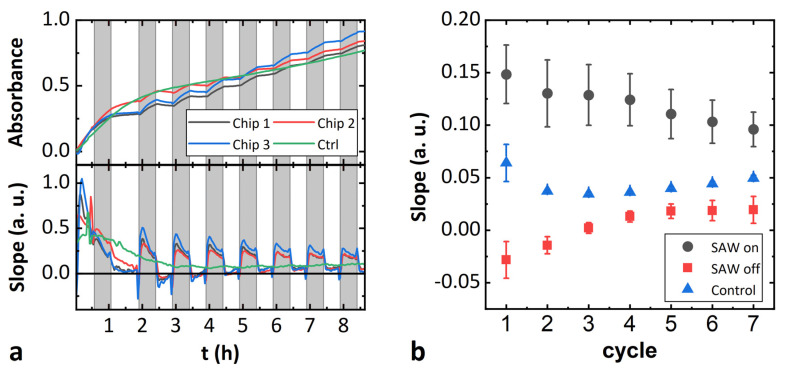
Influence of a SAW standing wave field on enzyme activity. (**a**) Time evolution of the absorbance and the first derivative thereof. A SAW is applied during the gray-shaded time intervals. (**b**) Time-averaged slope for the intervals with and without SAW treatment. Error bars represent the standard deviation of the three samples.

**Figure 6:**
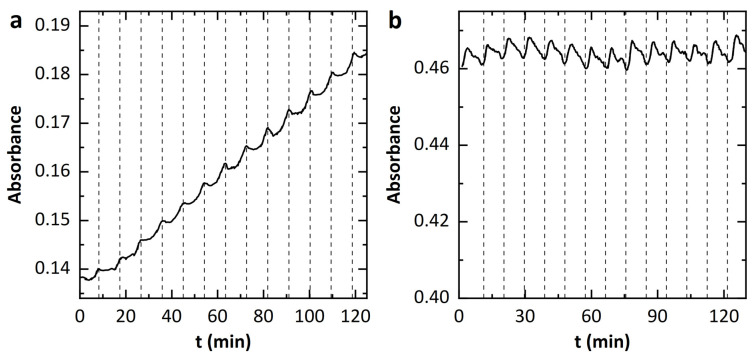
Influence of an alternating electric field on the absorption of TNB^2−^. (**a**) Measurement of the absorbance during the kinetic of AChE under the influence of an oscillating field with frequency sweep from 1 MHz to 80 MHz. (**b**) Measurement of absorbance for a saturated solution of the Ellman assay with the same electric field parameters.

In addition to the endpoint measurements under the influence of SAW, we also investigated the influence of SAW in enzyme kinetics. The samples were prepared in the same way as described above. To keep the heat input by the SAW as low as possible, a pulsed RF signal was used (f = 163.5 MHz, P = 20 dBm, cycle 50%, duration 30 min). The absorbance was then measured on a microscope (see materials and methods). Figure 5a shows the time course of the measured absorption and the first derivative with respect to time thereof for three separate samples with applied SAW and a control, which is identical to sample 1 in Figure 4b. At the very beginning of the kinetics, we observed that the kinetic is not yet linear due to slight inhomogeneities, that are equilibrated within the first hour. These inhomogeneities are probably due to the fact, that the substances were added together during the preparation of the solutions without extensive mixing to prevent the enzyme from being flushed from the membrane. Subsequently, the reaction is linear during the remaining time. The gray intervals indicate when a pulsed SAW was launched. In the other intervals, the RF signal was switched off. The increased slope in the time intervals with SAW application is clearly visible. This is particularly well seen in the first derivative of the signal with respect to time. Abnormally, there is a short drop in the signal immediately after the RF signal is turned on. In Figure 6b, the time-averaged slopes are plotted over the time intervals. It is obvious that the slope is always higher in the time intervals with SAW than in the intervals without SAW. However, this difference between the two regions becomes smaller with time. It is also unexpected here that the slope for intervals without SAW is negative at the beginning. This result shows that the SAW treatment has a clear effect on the measured absorbance. However, it is not clear whether this effect is due to a change in enzyme activity. It is more likely that the behavior shown is due to interfering effects on the absorption of the dye induced by the SAW. These effects might superimpose any effects of SAW-induced membrane area fluctuations on enzyme activity here.

### 3.4. Influence of an Electric Field on Enzyme Activity

A possible interfering effect of the SAW on the absorption behavior could originate from the electric field accompanying the SAW. To test the influence of an alternating electric field on the absorption separately, a sample chamber made of a PTFE ring was used. The PTFE ring is sealed on both sides with two ITO-coated glass plates (see Materials and Methods). A radio-frequency alternating voltage is applied to the conductive glass plates. The generated electric field is E = 8 V/cm. Figure 6a shows an example of an absorption measurement with the RF signal turned on. The signal was swept between 1–80 MHz with a period of 500 s. Overall, the absorbance increases linearly with time. The periodic fluctuations of the absorption are striking. The time between two of these peaks corresponds to the period of a frequency sweep. It is questionable whether these fluctuations are due to changes in enzyme activity, since the absorbance initially decreases briefly after each of the peaks. This means that the dye concentration in the sample would have to decrease shortly due to a reverse reaction. Alternatively, the extinction coefficient could also be affected by the electric field. To check whether this result is an artifact, a saturated sample was examined under the same AC electric field. The result of this experiment is shown in Figure 6b. A periodic variation of the absorbance also occurs in the saturated sample. Again, the time interval between two maxima is equal to the sweep duration. These similarities to the measurement of kinetics suggest that an artifact is the cause of the variation. However, it cannot be excluded that the artifact superimposes an actual small change in enzyme activity.

### 3.5. Decay of Domains

**Figure 7:**
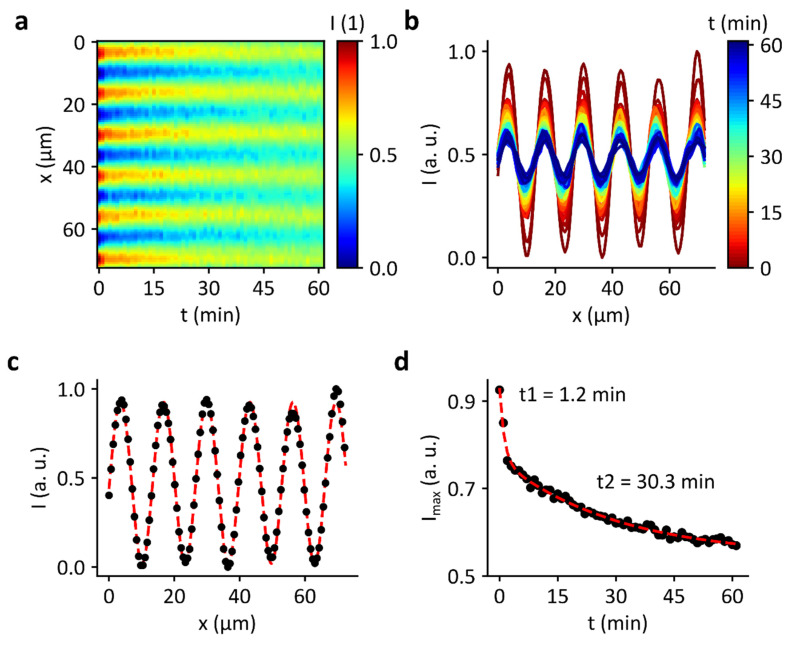
Domain decay in an SLB after switching off the RF signal inducing a SAW standing wave field. (**a**) Heatmap of the normalized brightness intensity over a period of 60 min. (**b**) Intensity profile perpendicular to the pattern of stripes. (**c**) Intensity course for t = 0 min with fit function according to Equation (5). (**d**) Dependence of the intensity maximum as a function of time after switching off the SAW. The intensity decreases with time. However, the stripe pattern is still visible after one hour.

Another aspect that could play a role in the influence of the standing wave field on enzyme activity is whether the SLB is intact. Therefore, as a control we investigated the decay of the domains when switching of the RF signal and thus the compressing force field. Figure 7a shows the normalized and smoothed brightness intensity after the RF signal (f = 163.5 MHz and P_IDT_ = 27 dBm) was turned off. After 60 min, the pattern of stripes is still clearly visible. Figure 7b shows the intensity profile perpendicular to the striped pattern. The color code corresponds to the time course. After 60 min, the sinusoidal intensity between the bright and dark areas is also still present. Figure 7d shows the time course of the intensity maximum (extracted from the fitted profile in Figure 7c) and thus the decay of the domains. Two timescales can be observed (t1 = 1.2 min and t2 = 30.3 min). Hennig et al. also reported that domain decay occurs with two decay rates, resulting in t1 = 9 s and t2 = 90 s [12]. They ascribe this phenomenon to strong differences in the viscosity of bulk water and surface-bound nanoscopic water layers or potentially induced nanoscopic membrane defects that heal after the SAW has been switched off [12]. However, the decay times in our experiment are much higher. One explanation for this discrepancy could be that the bilayers presented by Hennig et al. contained different materials (such as soybean extract, CTAB and TexasRed-labelled DHPE). Yet, the result indicates that the reversibility of the formation and decay of the domains is strongly limited in our experiments. This could be an explanation for the fact that the enzyme is not affected by the standing SAW field.

**Figure 8:**
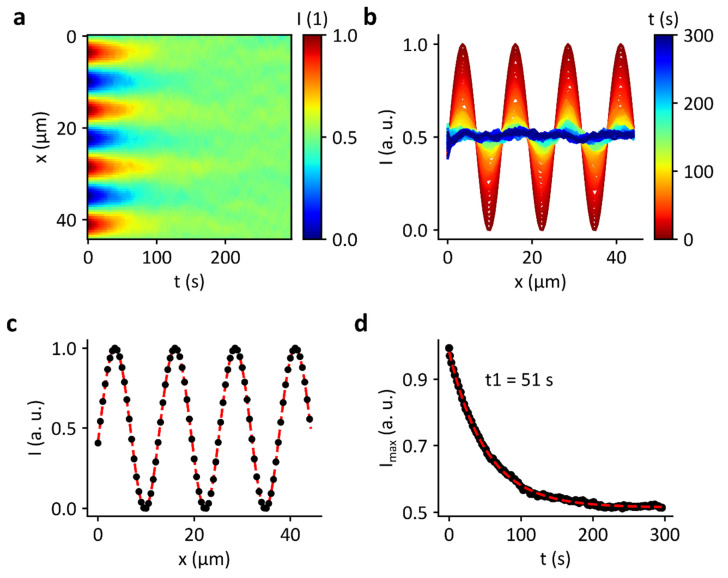
Simulation of domain decay in an SLB. (**a**) Heatmap of the normalized brightness intensity over 300 s. (**b**) Intensity profile perpendicular to the pattern of stripes. (**c**) Fitted intensity curve for t = 0 s. (**d**) Time course of the intensity maximum of the fit curves. After about 100 s, the stripe pattern has almost completely disappeared.

The discrepancy between the results of Neumann and Hennig et al. and those obtained in this work was examined in more detail using a simulation of the domain decay. Figure 8a shows the normalized brightness intensity over 300 s as a heatmap for a simulation with 100 × 100 grid points and a diffusion constant of 0.1 μm^2^/s, which was observed in the experiment shown in Figure 7. At the beginning, the given sinusoidal pattern of stripes is clearly visible. With increasing time, the pattern disappears completely. This can also be seen in Figure 8b. Here, the intensity curve is shown perpendicular to the stripes. After approximately 100 s, the pattern is hardly seen anymore. The decay of the pattern can also be visualized by plotting the intensity maximum (extracted from the fits in Figure 8c) as function of time. Here, only a single exponential decay can be detected. This is not surprising as only a single layer, or leaflet, is simulated.

The first part of our study presented here indicates that it is technically challenging to induce membrane density fluctuations in a lipid membrane with surface acoustic waves and measure the activity of embedded enzymes at the same time. Nevertheless, we cannot fully exclude an effect of dynamic area fluctuations on AChE activity as that might be superimposed by the artifact signals of the electric field. In the following, we therefore proceed to a simplified more static experimental setup, where we alter the available membrane area per enzyme on small unilamellar vesicles. As presented in detail in the next section, this will help us to understand if the artificial reduction of membrane area has any influence on the activity of embedded enzymes, as suggested in previous works [21,22,23].

### 3.6. Enzyme Activity Bound to Lipid Membranes

**Figure 9:**
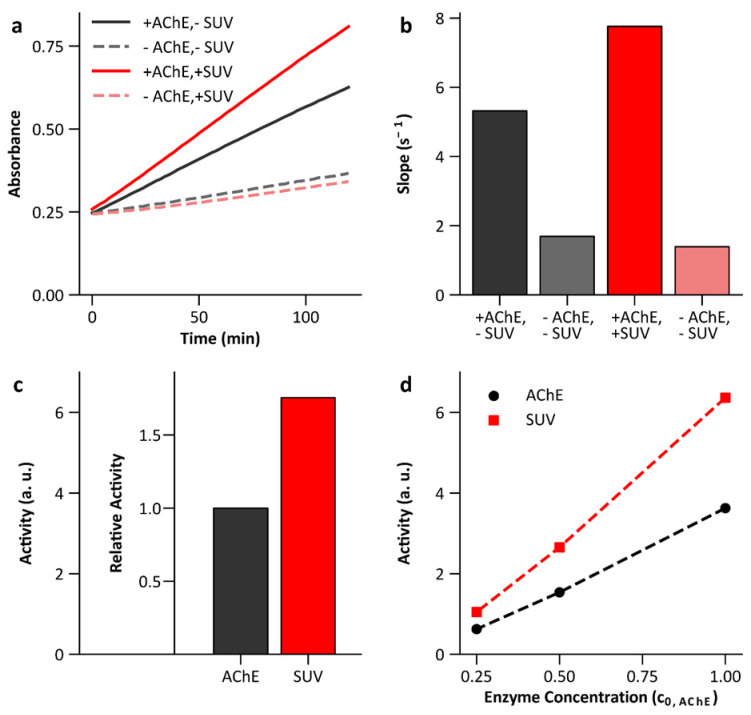
Enzyme activity of AChE in presence and absence of lipid membranes. (**a**) Absorbance measurement at room temperature using the Ellman assay. (**b**) Slope of the absorption kinetics determined by a linear fit. (**c**) The activity results from the difference of the slopes of the samples with and without enzymes. (**d**) Activity in dependence of the enzyme concentration with and without SUV (c_0,AChE_ = 0.2 nm).

**Figure 10:**
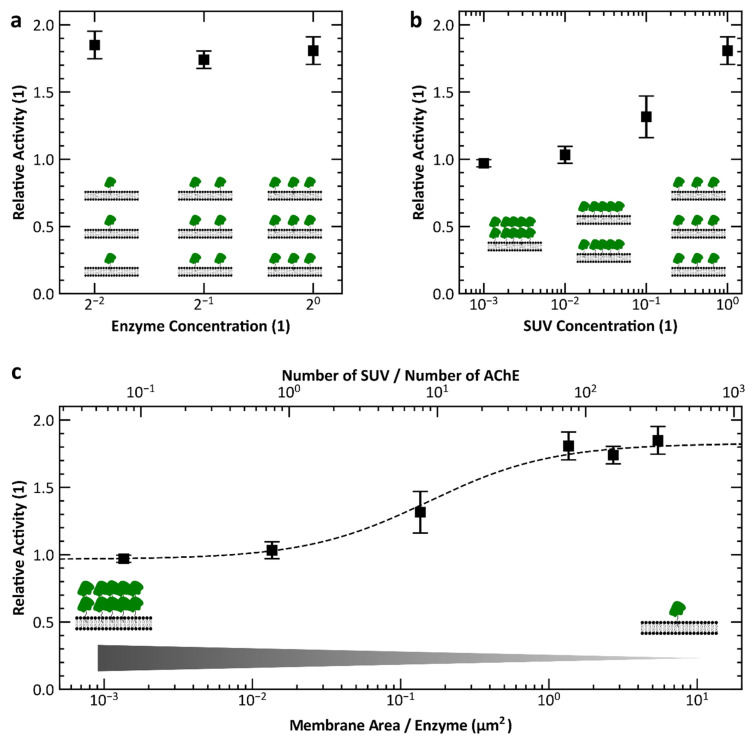
Influence of the available lipid membrane area on enzyme activity. (**a**) At a lipid concentration of 1 mg/mL, there is a significant increase in enzyme activity compared to the free enzyme (relative activity = activity with lipid/activity free enzyme). This effect is independent of the enzyme concentration. (**b**) If the lipid concentration is reduced, the effect decreases. At a 1000-fold reduction in lipid concentration, increased enzyme activity is no longer observed. (**c**) Relative activity as a function of available membrane area per enzyme calculated from (**a**,**b**). Error bars represent the standard error of the mean, n = 3.

As we have shown, the activity of a membrane-bound enzyme is not significantly affected by the SAW compared to electrically induced artifacts when it is bound to an SLB. This could be due to the fact that the change in membrane area caused by the SAW in our experiments is rather limited and due to the long-time constants observed, is not reversible. However, it does not exclude the idea that there is a relationship between the enzyme activity and the available membrane area. Therefore, measurements of AChE activity with and without lipid were performed in addition. SUV are used as a model system as they are much easier to handle than an SLB. Figure 9 shows one representative study with the aim to illustrate how the results in Figure 10 are obtained. Figure 9a shows the measurement of absorbance using the Ellman assay for a sample with and without DOPC SUV (c_0,AChE_ = 0.2 nM, c_0,DOPC_ = 0.2 mM). During the period considered, both samples show a linear trend. The absorbance of the sample with lipid is significantly increased. The controls do not contain the enzyme and thus represent the thermal decay of ASCh, which is comparable for both samples. To determine the activity from the absorption kinetics, the slope is first determined by a linear fit (Figure 9b). The activity is then obtained from the difference between the slopes of the sample and the control, since this is proportional to the product formed by the enzymes. Figure 9c shows the activity of the two samples in comparison. It is clearly increased for the sample with lipid. Figure 9d shows the dependence of the activity on the enzyme concentration. This is given in units of c_0,AChE_. A linear dependence can be seen for both the samples with and without lipid.

These results show that the presence of a lipid membrane has a considerable influence on the activity of AChE. This relationship is elucidated further by varying the amount of available membrane area per enzyme. This can be done by varying the enzyme concentration. Figure 10a shows the relative activity as a function of enzyme concentration. For the three concentrations considered, the relative activity is approximately constant at about the relative activity ≈ 1.9. Thus, the activity of the lipid-bound enzyme is almost twice as high as the activity of free enzyme. Decreasing the enzyme concentration increases the lipid to enzyme ratio. However, since the increase in activity does not increase further, it can be assumed that the effect is in saturation. It would still be interesting to examine what happens when the lipid to enzyme ratio is decreased. However, the enzyme concentration can only be varied within a limited range. Too high concentrations will cause the reaction to proceed very rapidly making it difficult to ensure that the experiment takes place in the linear range of the kinetics. A decrease in concentration, on the other hand, causes the reaction to proceed more slowly and one approaches the value obtained by thermal decay of ASCh. Therefore, in a further step, the lipid concentration was decreased, leading to a decrease in the lipid to enzyme ratio. Figure 10b shows that with increasing lipid concentration, the relative activity also increases. At the lowest concentration, the relative activity is 1, which corresponds to the activity of the free enzyme. This result confirms the assumption that the lipid to enzyme ratio is crucial here. The potentially concentration-dependent partition coefficient of the enzyme, between membrane and bulk, might additionally contribute to the effects observed here. An explanatory approach is offered by considering the available membrane area per enzyme. This can be calculated from the lipid and enzyme concentration (see Figure 10c).

## 4. Conclusions

First, we investigated the influence of a standing acoustic wave field on the activity of AChE bound to a SLB with the intention to dynamically modulate the enzyme density in the membrane. We were able to show that there was no effect on the enzyme activity. This could be due to the fact that the standing acoustic wave field causes only a small change in the available membrane area. Moreover, in contrast to previous studies, the density modulation of the SLB was not reversible in our experiments. Nonetheless, we demonstrated that there is a strong effect of lipid membranes on the activity of AChE by using DOPC SUV as a model system. We also showed that the effect scales with the available membrane area per enzyme. When the membrane area per enzyme is less than 10^−3^ μm^2^, the activity corresponds to the activity of the lipid-free enzyme. For values of membrane area per enzyme greater than 10^1^ μm^2^, the effect saturates, and the activity is twice the lipid-free enzyme activity. These findings could contribute to discussing the role of heterogeneous cell membranes with coexistence of phases with varying order and/or composition especially in the light of order–disorder phase transitions in biologicals membranes [38] or signal propagation at biological interfaces [39].
